# ICTV Virus Taxonomy Profile: *Bornaviridae*

**DOI:** 10.1099/jgv.0.001613

**Published:** 2021-07-06

**Authors:** Dennis Rubbenstroth, Thomas Briese, Ralf Dürrwald, Masayuki Horie (堀江真行), Timothy H. Hyndman, Jens H. Kuhn, Norbert Nowotny, Susan Payne, Mark D. Stenglein, Keizō Tomonaga (朝長啓造), ICTV Report Consortium

**Affiliations:** ^1^​ Institute of Diagnostic Virology, Friedrich-Loeffler-Institut, Greifswald – Isle of Riems, Germany; ^2^​ Center for Infection and Immunity and Department of Epidemiology, Mailman School of Public Health, Columbia University, New York, USA; ^3^​ Robert Koch Institute, Berlin, Germany; ^4^​ Hakubi Center for Advanced Research, Kyoto University, Kyoto, Japan; ^5^​ School of Veterinary Medicine, Murdoch University, Murdoch, Western Australia, Australia; ^6^​ NIH/NIAID/DCR/Integrated Research Facility at Fort Detrick, Frederick, Maryland, USA; ^7^​ University of Veterinary Medicine Vienna, Vienna, Austria; ^8^​ Mohammed Bin Rashid University of Medicine and Health Sciences, Dubai, UAE; ^9^​ Department of Veterinary Pathobiology, College of Veterinary Medicine and Biomedical Sciences, Texas A&M University, College Station, Texas, USA; ^10^​ Colorado State University, Fort Collins, Colorado, USA; ^11^​ Institute for Frontier Life and Medical Sciences (inFront), Kyoto University, Kyoto, Japan

**Keywords:** ICTV Report, *Mononegavirales*, *Bornaviridae*, *Orthobornavirus*, *Cultervirus*, *Carbovirus*, Borna disease virus, bornavirus

## Abstract

Members of the family *Bornaviridae* produce enveloped virions containing a linear negative-sense non-segmented RNA genome of about 9 kb. Bornaviruses are found in mammals, birds, reptiles and fish. The most-studied viruses with public health and veterinary impact are Borna disease virus 1 and variegated squirrel bornavirus 1, both of which cause fatal encephalitis in humans. Several orthobornaviruses cause neurological and intestinal disorders in birds, mostly parrots. Endogenous bornavirus-like sequences occur in the genomes of various animals. This is a summary of the International Committee on Taxonomy of Viruses (ICTV) Report on the family *Bornaviridae*, which is available at ictv.global/report/bornaviridae.

## Virion

**Fig. 1.**
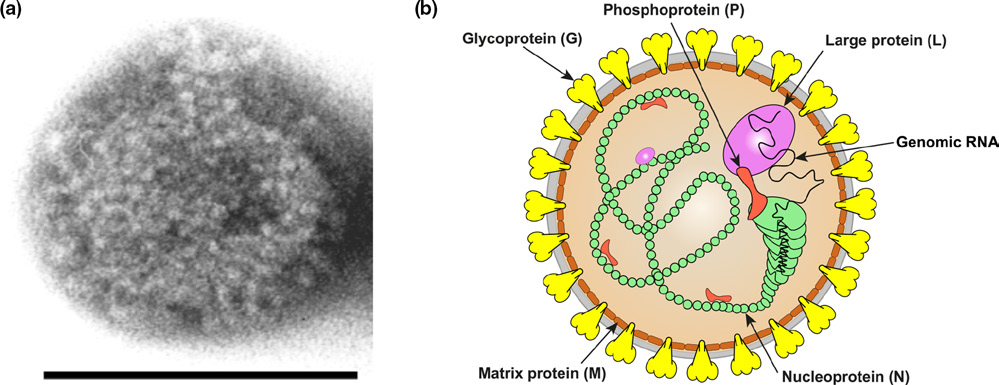
(a) Electron micrograph of a Borna disease virus 1 particle. Scale bar, 100 nm. Courtesy of Dr M. Eickmann. (b) Illustration of an orthobornavirus particle. Grey - bilaminar lipid envelope.

Where known, bornavirus virions are spherical in shape with a bimodal size distribution of larger (110–130 nm diameter) and smaller particles (70–90 nm diameter) [1, 2]. Virions are enveloped with 7-nm spikes and are believed to bud from host cell membranes (Table 1, Fig. 1). Entry occurs through binding to unknown cell surface receptors via the endosomal route.

**Table 1.** Characteristics of members of the family *Bornaviridae*

Example:	Borna disease virus 1 (U04608), species *Mammalian 1 orthobornavirus*, genus *Orthobornavirus*
Virion	Enveloped, spherical virions 90–130 nm in diameter
Genome	Linear negative-sense non-segmented RNA of about 9 kb with three transcription units and at least six ORFs
Replication	Intranuclear. Anti-genomic RNA is generated as a replication intermediate that enables synthesis of progeny genomes. Genomic and anti-genomic RNA molecules are neither capped nor polyadenylated
Translation	From capped polyadenylated mRNA
Host range	Mammals (reservoir: shrews and squirrels; incidental: horses, sheep, humans and other mammals), birds (parrots, finches, aquatic birds), reptiles and fish
Taxonomy	Realm *Riboviria*, kingdom *Orthornavirae*, phylum *Negarnaviricota*, subphylum *Haploviricotina*, class *Monjiviricetes*, order *Mononegavirales*; several genera and >10 species

## Genome

The bornavirus genome consists of a linear negative-sense non-segmented RNA with six open reading frames (ORFs) in the order 3′-*N-X/P-M-G-L*-5′ (*Orthobornavirus*) or 3′-*N-X/P-G-M-L*-5′ (*Carbovirus* and *Cultervirus*) [3–6] that encode, at a minimum, a nucleoprotein (N), small accessory protein (X), phosphoprotein (P), matrix protein (M), surface glycoprotein (G) and large protein (L) containing RNA-directed RNA polymerase (RdRP), helicase and endonuclease domains.

## Replication

**Fig. 2.**
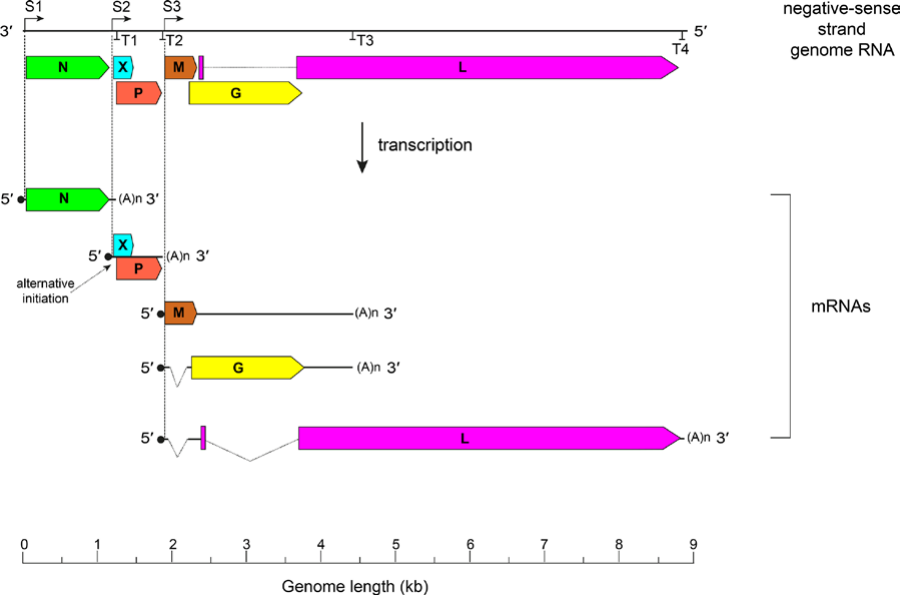
Orthobornavirus genome organization and transcripts. Dashed lines - introns. ORFs (depicted on the negative-sense strand) encode N, nucleoprotein; X, accessory protein; P, phosphoprotein; M, matrix protein; G, glycoprotein; L, large protein containing an RdRP domain. S, transcription initiation site; T, transcription termination site.

Studies of Borna disease virus 1 indicate that N encapsidates the genomic RNA, forming the viral nucleocapsid, and, together with L and P, forms the ribonucleoprotein (RNP) complex [3]. The genome is transcribed and replicated in the host cell nucleus [7]. Differential use of transcription and termination signals and alternative splicing of polycistronic primary transcripts generate an array of mRNAs (Fig. 2) [8]. Full-length antigenomic positive-sense RNA intermediates are used as templates for generating progeny negative-sense genomic RNA. Nuclear import of viral RNP components after translation of viral mRNA is mediated by their nuclear localization signals; nascent RNPs probably translocate into the cytosol from the nucleus by nuclear export signals [9].

## Taxonomy

Current taxonomy: www.ictv.global/taxonomy. Bornaviruses form a family in the haploviricotine order *Mononegavirales,* most closely related to members of the families *Mymonaviridae*, *Nyamiviridae* and *Xinmoviridae*. Like most other mononegaviruses, bornaviruses (i) have linear negative-sense non-segmented RNA genomes, (ii) have five conserved motifs (A–E) in the amino-acid sequence of their RdRP domain and (iii) produce enveloped virions.

## Resources

Current ICTV Report on the family *Bornaviridae*: www.ictv.global/report/bornaviridae.
